# DACT1 Involvement in the Cytoskeletal Arrangement of Cardiomyocytes in Atrial Fibrillation by Regulating Cx43

**DOI:** 10.21470/1678-9741-2019-0033

**Published:** 2019

**Authors:** Jian Hou, Yuan Yue, Bo Hu, Guangtao Xu, Ruibing Su, Linhua Lv, Jiaxing Huang, Jianping Yao, Yuanjun Guan, Keke Wang, Zhongkai Wu

**Affiliations:** 1 Department of Cardiac Surgery, The First Affiliated Hospital of Sun Yat-Sen University, Guangzhou, GD, People’s Republic of China.; 2 NHC Key Laboratory of Assisted Circulation, Sun Yat-Sen University, Guangzhou, GD, People’s Republic of China.; 3 Department of Pathology and Molecular Medicine Center, Jiaxing Hospital of Traditional Chinese Medicine, Jiaxing University, Jiaxing, ZJ, People’s Republic of China.; 4 Department of Forensic Pathology, Shantou University Medical College, Shantou, GD, People’s Republic of China.; 5 Core Lab Plat for Medical Science, Zhongshan School of Medicine, Sun Yat-Sen University, Guangzhou, GD, People’s Republic of China.

**Keywords:** Atrial Fibrillation, Myocardium, Cytoskeleton, Actins, Myocytes, Cardiac, Connexin 43, Flow Cytometry, Western Blotting

## Abstract

**Objective:**

To determine the role of the dishevelled binding antagonist of beta catenin 1 (DACT1) in the cytoskeletal arrangement of cardiomyocytes in atrial fibrillation (AF).

**Methods:**

The DACT1 expression and its associations with the degree of fibrosis and β-catenin in valvular disease patients were analyzed by immunohistochemistry and Masson’s staining. DACT1 was overexpressed in the atrial myocyte cell line (HL-1) and the cardiac cell line (H9C2) by adenoviral vectors. Alterations in the fibrous actin (F-actin) content and organization and the expression of β-catenin were detected by flow cytometry, immunofluorescence, and Western blotting. Additionally, the association of DACT1 with gap junctions connexin 43 (Cx43) was detected by immunohistochemistry, immunofluorescence, and Western blotting.

**Results:**

Decreased cytoplasmic DACT1 expression in the myocardium was associated with AF (*P*=0.037) and a high degree of fibrosis (weak *vs*. strong, *P*=0.028; weak *vs*. very strong, *P*=0.029). A positive association was observed between DACT1 and β-catenin expression in clinical samples (*P*=0.028, Spearman’s rho=0.408). Furthermore, overexpression of DACT1 in HL-1 and H9C2 cells induced an increase in β-catenin and subsequent partial colocalization of DACT1 and β-catenin. In addition, F-actin content and organization were enhanced. Interestingly, DACT1 was positively correlated with the Cx43 expression in clinical samples (*P*=0.048, Spearman’s rho=0.370) and changed the Cx43 distribution in cardiac cell lines.

**Conclusion:**

DACT1 proved to be a novel AF-related gene by regulating Cx43 via cytoskeletal organization induced by β-catenin accumulation in cardiomyocytes. DACT1 could thus serve as a potential therapeutic marker for AF.

**Table:** 

Abbreviations, acronyms & symbols				
AF	= Atrial fibrillation		LA	= Left atrial
AI	= Aortic insufficiency		LVD	= Left ventricular diastolic
AR	= Aortic regurgitation		LVPW	= Left ventricular posterior wall
AS	= Aortic stenosis		LVS	= Left ventricular systolic
B	= Beta-blockers		M	= Male
cDNA	= Complementary deoxyribonucleic acid		MOI	= Multiplicity of infection
Cx43	= Connexin 43		MI	= Mitral insufficiency
D	= Digoxin		MS	= Mitral stenosis
DACT1	= Dishevelled binding antagonist of beta catenin 1		N	= Nitrates
DAPI	= 2-(4-Amidinophenyl)-6-indolecarbamidine dihydrochloride		NYHA	= New York Heart Association
DI	= Diuretics		PBS	= Phosphate-buffered saline
DMEM	= Dulbecco's Modified Eagle Medium		PFA	= Paraformaldehyde
ECG	= Electrocardiogram		PVDF	= Polyvinylidene difluoride
EF	= Ejection fraction		RA	= Right atrial
F	= Female		RV	= Right ventricular
F-Actin	= Fibrous actin		SACs	= Stretch-activated channels
FBS	= Fetal bovine serum		SDS-PAGE	= Sodium dodecyl sulfate polyacrylamide gel electrophoresis
GAPDH	= Glyceraldehyde-3-phosphate dehydrogenase		SPSS	= Statistical Package for the Social Sciences
GFP	= Green fluorescent protein		SR	= Sinus rhythm
GSK3-β	= Glycogen synthase kinase beta		TI	= Tricuspid insufficiency
ICa,L	= L-type Ca^2+^ current		TS	= Tricuspid stenosis
IVS	= Interventricular septum			

## INTRODUCTION

Atrial fibrillation (AF) is a ubiquitous arrhythmia and causes considerable morbidity and mortality; however, its mechanism remains unclear. AF is known to be caused by significant atrial electrical and structural remodeling^[1]^. The cytoskeleton plays an important role in this process, because of its involvement in the channel opening mediated by integrin in the process of the force transmitting force indirectly to stretch-activated channels (SACs)^[2]^, as well as the regulation of L-type Ca^2+^ current (ICa,L) and signaling pathway transduction^[3]^. Moreover, the force transmission can be directly or indirectly mediated by the focal proteins on the cytoskeleton^[4]^. Acute mechanical changes produce electrophysiological alterations and arrhythmias, mediated by both the cytoskeleton and the extracellular matrix^[4-6]^. Once AF is induced, rapidly and inhomogeneously contracting and interacting atrial segments would tend to perpetuate electrophysiological dispersion^[4]^, which could facilitate the geometry (fibrosis and scarring of atrium) formation^[7]^. Thus, the cytoskeleton could be an essential systematic regulator and a potential therapy marker for AF.

β-catenin is not just an important transcriptional effector of the canonical Wnt signaling pathway in the nucleus, but a crucial regulator of the cytoskeleton and signal transduction outside the nucleus as well^[8]^. β-catenin can affect both the cellular microtubule arrays by interacting with the complex of EB1/P150/dynein/dynactin and P120-catenin and affect the organization of the cellular actin filament by interacting with actin-bundling protein^[9,10]^. In cardiac cells, β-catenin directly binds to the C-terminal region of cadherin, whereas α-catenins link the cadherin/catenin complex to the actin cytoskeleton^[11]^. Thus, β-catenin is essential for the maintenance of cardiac cellular function. Abnormal β-catenin *in vivo* could lead to hypertrophic cardiomyopathy, heart failure, and arrhythmias^[12,13]^. However, the mechanism of cytoskeletal arrangement via β-catenin involvement in AF remains unclear.

A previous study showed that the dishevelled binding antagonist of beta catenin 1 (DACT1), a novel bidirectional regulator of β-catenin, was associated with cytoplasmic β-catenin accumulation, suggesting its potential role in cytoskeletal arrangement^[14]^. Yuan et al.^[15]^ suggested that DACT1 induced the β-catenin accumulation in the cytoplasm by inhibiting the glycogen synthase kinase beta (GSK3-β) activity and interacting directly with β-catenin. The actin cytoskeleton, which may be regulated by β-catenin, serves as a link to the adherens junctions, gap junctions, and desmosomes, which constitute the intercalated disc^[16]^. The remodeling of gap junctions, which are assembled from connexins, has been acknowledged to be an important contributor to AF^[17]^. Thus, we hypothesized that a new mechanism mediated by DACT1 might exist by which the stability of gap junctions is regulated in AF. However, little is known about this mechanism.

In the present study, we demonstrated that DACT1 is involved in AF. Then, we demonstrated that DACT1 was involved in AF by regulating the actin cytoskeleton. Furthermore, we found out that connexin 43 (Cx43) remodeling might be the result of the effect of DACT1 on the cytoskeleton in AF.

## METHODS

### Ethics

This study was approved by the Human Ethics Committee of the First Affiliated Hospital of Sun Yat-Sen University and complied with the principles governing the use of human tissues that are outlined in the Declaration of Helsinki. Informed consent was given by patients before participating in the study.

### Human Tissue Preparation

Tissue samples from the right atrial (RA) appendage were obtained from 29 patients with valvular heart disease who had undergone valve replacement surgery. Ten patients, who constituted the sinus rhythm (SR) group, did not have a history of AF, and 19 patients, who constituted the AF group, had documented arrhythmias from which they had suffered for more than six months before undergoing surgery. These tissue samples were obtained during the surgeries, before the aortic cross-clamping, and were immediately fixed in 4% paraformaldehyde (PFA). The diagnosis of AF was made based on patient medical records and 12-lead electrocardiogram (ECG) findings. All subjects underwent ECG to verify the underlying SR and an echocardiogram finding to document the preserved valvular and myocardial function. Preoperative functional statuses were recorded in accordance with the New York Heart Association (NYHA) classification. Patients’ data are summarized in Supplement Table 1.

**Supplement Table 1:** Patient's characteristics.

Age	Sex	VD	LA (mm)	LVD (mm)	LVS (mm)	IVS (mm)	LVPW (mm)	RA (mm)	RV (mm)	EF (%)	NYHA	Drug therapy	CHADS2 score	CHA2DVASc
**SR (n=10)**														
31	M	MS, MI, AI	50	51	30	9	9	46	25	75	2	D, DI	0	0
66	M	MS, MI, TI, AR, AS	36	52	35	11	10	45	52	62	2	A, D, DI	1	1
66	F	MI, AS, AI	37	52	36	13	10	43	19	58	2	D, DI	1	2
51	M	MI, AI	37	58	34	9	10	45	22	70	2	A, D, DI	1	2
43	F	MS, MI, TI, AS, AI	51	46	31	8	8	49	18	62	2	D, DI	0	1
65	M	MS, MI, TS, TI, AI	47	45	30	9	9	70	25	63	3	A, D, DI	2	2
57	M	MI, AS, AI	31	69	50	14	13	36	20	53	3	A, D, DI, N	0	0
71	F	MI, AS, AI	38	53	37	14	12	41	19	55	2	D, DI	1	2
37	F	TI	45	35	22	8	11	46	17	68	2	D, DI	2	3
58	M	MS, MI, TI, AI	55	51	31	8	8	51	24	66	2	D, DI, B	0	0
**TOTAL**														
54.5±13.618	6M/4F		42.7±7.931	51.2±8.791	33.6±7.168	10.35±2.450	10±1.633	47.2±9.016	24.1±10.225	63.2±6.812	2.2±0.422		0.8±0.789	1.2±1.033
**AF(n=19)**														
58	F	MS, MI, TI, AI	85	57	39	7	9	72	20	67	3	D, DI	0	1
52	F	MS, MI, TI, AI	58	54	34	11	9	64	64	69	2	DI	2	3
46	F	MS, MI, TI, AS, AI	63	55	38	12	9	85	28	55	3	D, DI, B	0	1
34	F	MS, MI, TI, AI	122	63	38	8	8	37	21	66	2	D, DI, B	0	1
48	F	MS, MI, TI, AI	50	44	27	11	10	60	24	60	3	A, D, DI, C	1	2
54	F	MS, MI, TI, AI	89	40	26	9	10	59	26	54	3	D, DI	0	1
59	M	MS, TI	51	49	34	9	10	52	25	56	3	D, DI	0	0
		AI												
55	F	MS, MI, TS, TI, AS, AI	51	32	24	9	10	79	31	60	2	D, DI	0	1
46	F	MS, MI, AI	42	41	29	9	8	61	23	62	3	DI	0	1
62	M	MS, MI, TI, AI	40	48	34	8	7	64	26	79	3	D, DI	2	2
48	M	MI, MS, TI, AI	46	48	33	10	9	60	21	64	2	D, DI	0	0
48	F	MS, MI, TI, AS, AI	57	42	28	11	9	57	20	76	4	DI	0	1
60	F	MI, TI, AI	98	66	34	11	11	86	29	54	2	D, DI, N	0	1
32	M	MI, TI, AI	55	61	39	9	9	56	25	63	3	D, DI	0	0
56	M	MS, MI	82	65	35	10	10	67	23	69	2	D, DI, N	0	0
52	F	MI, TI, AI	51	65	46	13	13	62	21	62	2	D, DI, B	3	4
24	F	MS, MI, TI, AI	60	64	41	9	12	44	21	67	3	D, DI	.00	1.00
60	M	MI, TI, AI	44	63	38	9	9	69	24	69	2	DI	.00	.00
56	F	MS, MI, TI, AS, AI	60	45	30	7	8	70	24	55	3	D, DI	.00	1.00
**TOTAL**														
50±10.301	6M/13F		63.368±21.843*	52.737±10.402	34.053±5.690	9.579±1.610	9.474±1.429	63.368±12.321*	26.105±9.678	62.526±7.597	2.65±0.671*		0.421±0.902	1.105±1.049

* P<0.05 vs. SR.

A=angiotensin-converting enzyme inhibitors; AF=atrial fibrillation; AI=aortic insufficiency; AR=aortic regurgitation; AS=aortic stenosis; B=beta-blockers; C=calcium-channel blockers; D=digoxin; DI=diuretics; EF=ejection fraction; F=female; IVS=interventricular septum; LA=left atrial; LVD=left ventricular diastolic; LVPW=left ventricular posterior wall; LVS=left ventricular systolic; M=male; MI=mitral insufficiency; MS=mitral stenosis; N=nitrates; NYHA=New York Heart Association; RA=right atrial; RV=right ventricular; SR=sinus rhythm; TI=tricuspid insufficiency; TS=tricuspid stenosis; VD=valve disease requiring valve replacement. Independent Student's t-test was used for comparisons between two groups

The RA appendages of normal hearts were obtained from autopsies (two cases) and provided by the Department of Forensic Pathology of Shantou University Medical College and the Department of Forensic Pathology of Jiaxing University Medical College. The ages of the both male patients were 19 and 23 years. The hearts obtained at autopsy were devoid of any abnormal findings and the causes of death were not heart related.

### Immunohistochemical Staining

All the samples were fixed in 4% PFA, embedded in paraffin, and stained with hematoxylin and eosin for routine histological examination. Immunohistochemical staining was performed on 4-µm-thick tissue sections. After deparaffinization and rehydration, all the sections were microwaved (10 min) in 0.01 mol/L sodium citrate buffer (pH 6.0) for antigen retrieval. To block endogenous peroxidase activity, we incubated the sections with 10% normal goat serum in phosphate-buffered saline (PBS) for 15 min at room temperature. Then, all the sections were incubated with a rabbit polyclonal antibody against DACT1 (1:100; Abcam), β-catenin (1:100; Cell Signaling Technology), or Cx43 (1:100; Sigma) overnight at 4°C. The slides were subsequently treated with a SuperPic Ture Polymer Detection Kit and Liquid DAB Substrate Kit (Zymed/Invitrogen, San Francisco, USA) and counterstained with hematoxylin, dehydrated, and mounted.

### Masson’s Trichrome Staining

The sections were stained with Masson’s trichrome for fibrosis quantification. For Masson’s trichrome staining, the sections were dewaxed with xylol (two dewaxing steps lasting 2 min each, followed by soaks in a series of graded alcohols, with concentrations ranging from 95% to 99%). All the slices were then washed in distilled water and placed in a hematoxylin solution for 3 min, after which a color change was induced with lithium carbonate. The slices were subsequently washed in pure water and colored with Ponceau red staining (in a 45 kW oven at 30°C for 20 sec). Next, the slices were placed in distilled water and phosphomolybdic acid for 1 min before being labeled with a green fluorescent marker and washed again with distilled water. The severity of fibrosis was then assessed in each of the sections upon their collection.

### Immunostaining Evaluation

Immunohistochemical expression was evaluated using the Image-Pro Plus 6.0 software (Media Cybernetics, Silver Spring, Maryland, USA). Briefly, at least three positive expression fields in a section of myocardial tissue were randomly selected, and then these positive regions were analyzed with the Image-Pro Plus 6.0 software to determine their integral optical density and area. The average of the optical density values, which represented the expression intensity in the section, was subsequently calculated.

### Fibrosis Evaluation

Fibrosis severity was evaluated using the Image-Pro Plus 6.0 software. At least three fields in a section of myocardial tissue were randomly selected after which the ratio of the fibrotic area to the total area of each selected field was calculated to assess fibrosis severity. The average ratio, which represented the severity of the fibrosis in the section of myocardial tissue, was subsequently determined.

### Replication-Defective Recombinant Adenoviral Vectors

Briefly, the complementary deoxyribonucleic acid (cDNA) of two transcript variant isoforms of DACT1 (named D1V1 and D1V2) were purchased from GeneCopoeia (cat: HOC23467 and GC-H1635, respectively), and inserted into pGA1-2A-GFP through enzymatic digestion and ligation to obtain pGA1-D1V1-GFP and pGA1-D1V2-GFP. The autocleavage 2A linker between DACT1 and green fluorescent protein (GFP) facilitates the formation of separated DACT1 and GFP reporter. Subsequently, pGA1-D1V1-GFP and pGA1-D1V2-GFP were linearized by enzymatic digestion and subjected to homologous recombination with the linearized pAd5∆E1∆E3 backbone in competent *E. coli* BJ5183 cells to obtain pAd5-D1V1-GFP and pAd5-D1V2-GFP, respectively. pAd5-D1V1-GFP and pAd5-D1V2-GFP were amplified in competent *E. coli* XLI-Blue cells and prepared with a Qiagen Plasmid Midi kit (QIAGEN), according to the manufacturer’s protocol. pAd5-D1V1-GFP and pAd5-D1V2-GFP were then linearized and transfected into HEK293 cells to rescue and propagate the respective recombinant adenoviruses. The expression levels of DACT1 were analyzed further by Western blotting analysis, and GFP-expressing cells were observed using a fluorescence microscope (Axio Observer Z1).

### Cell Culture and Adenovirus Infection

The H9C2 and HL-1 cell lines were used in this study. The HL-1 cell line was obtained from EMD Millipore Corporation (Cat. SCC065). Cells were cultured using Claycomb medium supplemented with 10% fetal bovine serum (FBS), 2 mM L-glutamine, 100 U/mL penicillin, and 100 mg/L streptomycin in flasks precoated with fibronectin and gelatin. The H9C2 cell line was cultured in Dulbecco's Modified Eagle Medium (DMEM)/high-glucose medium (HyClone) with 15% FBS and antibodies (100 U/mL penicillin and 100 mg/L streptomycin). All the cells were grown under a 5% CO_2_ atmosphere at 37°C.

Cells were plated 24 hours before transduction, incubated with fresh media containing the required multiplicity of infection (MOI) per cell of the virus, left for 18 hours, washed, and maintained until harvesting. Forty-eight hours after transduction, GFP-expressing cells were observed using a fluorescence microscope (Axio Observer Z1).

### Western Blotting

The technique used was described previously^[18]^. Proteins were isolated from cells with lysis buffer (Beyotime Institute of Biotechnology, Shanghai, China) that included a protease inhibitor cocktail (Millipore, Billerica, Massachusetts, USA). Proteins were subjected to sodium dodecyl sulfate polyacrylamide gel electrophoresis (SDS-PAGE) and transferred to polyvinylidene difluoride (PVDF) membranes (Millipore, Billerica, Massachusetts, USA). Primary antibodies against DACT1 (1:1000; Origene), β-catenin (1:1000; Cell Signaling Technology), or Cx43 (1:1000; Sigma) were used, and antigen-antibody complexes were detected by a Western blotting luminol reagent (Santa Cruz Biotechnology). Glyceraldehyde-3-phosphate dehydrogenase (GAPDH) (Proteintech) or β-actin (Wuhan GoodBio Tech. CO. LTD) served as an internal reference, and at least two independent experiments were performed for each cell line. Image J software was used to analyze the mean light density of each band. The expression of target genes was normalized to that of GAPDH or β-actin.

### Quantitation of Fibrous Actin (F-Actin) Levels

The technique used was described previously^[18]^. The F-actin analysis was conducted via flow cytometry, and cells were fixed with 70% ethanol overnight at 4°C. Cell pellets were incubated in PBS containing 0.1% Triton-X 100 for 15 min at room temperature and then they were treated with phalloidin-coumarin (2 mg/mL; Thermo Fisher) for 30 min. The F-actin content was analyzed by flow cytometry (Beckman, cytoFLEX). FlowJo 7.6 software (FlowJo, Ashland, Oregon, USA) was used to analyze the F-actin content in each group.

### Immunofluorescence Assay

The technique used was described previously^[18]^. Cells were fixed with 4% PFA for 15 min, blocked, and permeabilized with 1% FBS and 0.2% Triton X-100 in PBS at room temperature for one hour. Then, the cells were incubated with primary antibody overnight at 4°C. Primary antibodies against DACT1 (1:200; Origene), β-catenin (1:200; Cell Signaling Technology), or Cx43 (1:200; Sigma) were used at the indicated dilutions. After the cells were washed three times with PBS, secondary antibody (Alexa Fluor 488, Alexa Fluor 568, or Alexa Fluor 647) was diluted at 1:500 and applied to the cells for one hour at room temperature. For F-actin staining, the cells were incubated with 100 nmol/L Alexa Fluor® 647 Phalloidin (Thermo Fisher). Finally, the cells were incubated with 1µg/mL 4,6-diamidino-2-phenylindole (Sigma). Cells were analyzed by confocal fluorescence microscopy (Zeiss 780 NLO).

### Statistical Analysis

Correlations between the immunohistochemical results and patient clinical variables were analyzed by χ^2^-tests. Continuous variables are presented as the mean ± SEM. Comparisons of continuous variables between groups were performed with Student’s *t*-test, and the correlations between DACT1 expression levels and β-catenin or Cx43 were assessed with the nonparametric Spearman’s rank correlation test. *P*<0.05 was considered to indicate statistical significance. All statistical analyses were performed with Statistical Package for the Social Sciences (SPSS) software, version 19.0.

## RESULTS

### Association between DACT1 Expression and AF in Valvular Heart Disease

**Fig. 1:**
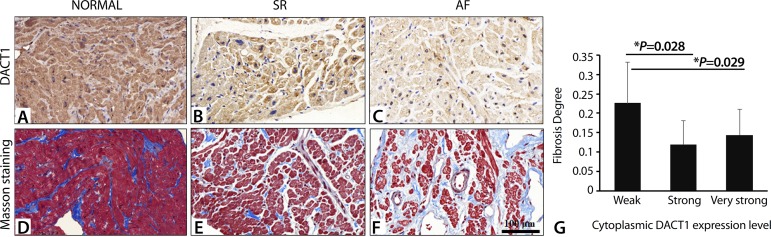
Immunohistochemical analysis of DACT1 expression in the myocardial tissues of patients with valvular heart disease. DACT1 expression was detected in the right auricular tissue of A) the normal heart, B) SR, and C) AF groups. Masson’s staining was used to observe the fibrosis severity in D) the normal heart, E) SR, and F) AF groups. G) Difference in patients’ degree of fibrosis between the weak and strong DACT1 expression groups (SR: n=10; AF: n=19). Fibrosis degree was expressed as the area of fibrosis/total area. AF=atrial fibrillation; DACT1=dishevelled binding antagonist of beta catenin 1; SR=sinus rhythm

DACT1 was located in both the cytoplasm and nucleus of cardiac cells in human myocardial tissue (Figures 1A, 1B, and 1C). Very strong staining for DACT1 was detected in the normal human heart tissue (Figure 1A), and decreased expression was observed in the myocardial tissue of the patients with valvular heart diseases (Figures 1B and 1C). The basic characteristics of patients with valvular diseases are summarized in Supplement Table 1. A significant difference was observed in left atrial (LA) diameter, RA diameter, and NYHA classification, while no significant difference was observed in CHADS2 score and CHA2DVASs between the AF and SR groups (Supplement Table 1). Furthermore, the relationship between DACT1 expression and clinical features was analyzed. In the SR group, no patient was detected to have weak staining for cytoplasmic DACT1 level, while in the AF group, the percentage was significantly increased, suggesting that a decrease in DACT1 was associated with AF (*P*=0.037, Supplement Table 2). The degree of fibrosis observed was higher in the weak cytoplasmic DACT1 expression group than in other groups (weak=0.226±0.106, strong=0.120±0.060, very strong=0.144±0.067; weak *vs*. strong, *P*=0.028; weak *vs*. very strong, *P*=0.029) (Figures 1D to 1G). These data suggested that decreased cytoplasmic DACT1 levels participated in AF and might serve as a risk factor.

**Supplement Table 2:** The relationship between DACT1 expression in the myocardium and the cardiac rhythm of patients with valvular heart disease.

	Cytoplasmic DACT1 expression^a^			*P*	Nuclear DACT1 expression^b^		*P*
	Weak	Strong	Very strong		Negative	Positive	
SR	0(0.0%)	3(10.3%)	7(24.1%)	0.037*	2(6.9%)	8(27.6%)	0.419
AF	6(20.7%)	2(6.9%)	11(37.9%)		6(20.0%)	13(44.8%)	

a As long as the sample contained cells with DACT1 cytoplasmic staining, the staining was regarded as weak if mean density was <0.025, strong if it was between ≥0.025 and <0.04, and very strong if it was ≥0.04;

b As long as the sample contained cells with DACT1 nuclear staining, the staining was regarded as positive if the positive cell count was >5% and negative if the mean density was <5%. Chi-square analysis was used to examine the relationships among categorical variables.

* *P*<0.05.

AF=atrial fibrillation; DACT1=dishevelled binding antagonist of beta catenin 1; SR=sinus rhythm

### DACT1 Induced Cytoskeletal Rearrangement in Myocardial Cells

**Fig. 2:**
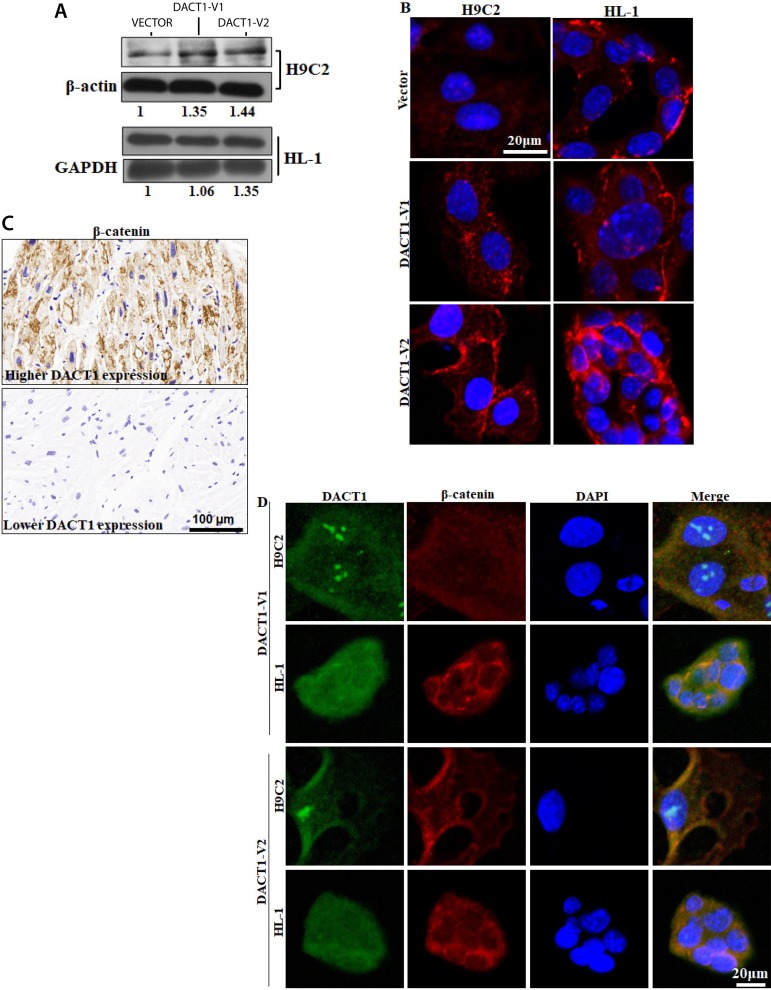
Effects of DACT1 on β-catenin in myocardial cells. Effect of DACT1 overexpression on β-catenin concentration A) and distribution B) in H9C2 and HL-1 cells. C) Correlation between DACT1 and β-catenin expression in the myocardial tissues of patients with valvular heart disease. D) Colocation of DACT1 and β-catenin in the myocardial cells. H9C2 and HL-1 cells were overexpressed with the two isoforms of the DACT1 coding sequence, and then immunofluorescence was used to detect the DACT1 (DACT1-V1 or DACT1-V2) (OriGene) and β-catenin (CST) location. The secondary antibody Alexa Fluor 647 was used for DACT1 detection and Alexa Fluor 568 for β-catenin. The ZEN (Zeiss) software was used for further analysis. Alexa Fluor 647 was represented by the green color and Alexa Fluor 568 by the red color, while channel 488 was closed to avoid interference. DACT1=dishevelled binding antagonist of beta catenin 1; DAPI=2-(4-amidinophenyl)-6-indolecarbamidine dihydrochloride; GAPDH=glyceraldehyde-3-phosphate dehydrogenase

In the present study, we investigated the regulatory role of DACT1 in β-catenin and cytoskeletal rearrangement. First, the endogenous expression of DACT1 was detected in the atrial myocyte cell line HL-1 and cardiac cell line H9C2, but neither of the cell lines was detected positive for DACT1 (Supplement Figure 1A). Thus, the HL-1 and H9C2 cell lines were selected for high exogenous DACT1 expression. The DACT1 gene has two alternatively spliced transcript variants with 111bp difference in exon4^[19]^; thus, we generated an adenovirus with two isoforms of DACT1 (named V1 or V2 in this study) for further investigation, and the overexpression efficiency was detected (Supplement Figures 1B and 1C). The concentration of β-catenin protein was just slightly increased after the two isoforms of DACT1 restored expression compared with that of the control group (Figure 2A). Immunofluorescence results showed that DACT1 could enhance the β-catenin staining in both HL-1 and H9C2 cells (Figure 2B), and further analysis in patient samples confirmed this finding. No significant association was observed between β-catenin expression and clinicopathological features (Supplement Table 3); however, we found a positive correlation between expression levels of DACT1 and β-catenin in patients with valvular heart disease (*P*=0.028, Spearman’s rho=0.408) (Supplement Table 4, Figure 2C). Moreover, both DACT1-V1 and DACT1-V2 displayed the partial colocalization with β-catenin in the HL-1 and H9C2 cells (Figure 2D). No DACT1 staining was observed in the vector group of HL-1 and H9C2 (data not shown). These results indicated that DACT1 could induce an alteration in β-catenin in the myocardial cell and might have functions in cytoskeletal rearrangement.

**Supplement Table 3:** Mean values comparing the clinical parameters of patients with valvular heart disease in different groups of β-catenin expression in the myocardium.

Indices	Weak	Strong	Very strong	*P*
EF (%)	63.1667±8.537	60.429±7.138	63.900±7.219	0.618
LA (mm)	58.000±25.856	45.857±10.961	61.400±17.702	0.300
LVD (mm)	54.000±9.737	50.429±11.886	50.700±8.564	0.583
LVS (mm)	35.083±6.037	33.714±8.180	32.600±4.835	0.213
IVS (mm)	10.333±2.146	10.428±1.989	8.850±1.292	0.131
LVPW (mm)	9.917±1.564	9.857±1.574	9.200±1.398	0.508
RA (mm)	57.000±16.321	53.571±14.397	61.700±8.932	0.479
RV (mm)	23.000±3.693	32.429±18.293	23.400±2.119	0.087
Fibrosis degree	0.160±0.109	0.139±0.066	0.164±0.053	0.817

As long as the sample contained cells with β-catenin cytoplasmic and/or membrane staining, the total expression level was regarded as negative if mean density was <0.01, positive if it was between ≥0.01 and <0.013, and strongly positive if it was ≥0.013. Fibrosis degree was detected by Masson's staining. Fibrosis degree=the area of fibrosis/total area. *P*-value comparison was performed between the three groups with analysis of variance. EF=ejection fraction; IVS=interventricular septum; LA=left atrial; LVD=left ventricular diastolic; LVPW=left ventricular posterior wall; LVS=left ventricular systolic; RA=right atrial; RV=right ventricular

**Supplement Table 4:** The relationship between DACT1 expression and β-catenin expression in the myocardium of patients with valvular heart disease.

β-catenin expression^b^	DACT1 expression^a^			*P* Spearman's rho
	Weak	Strong	Very strong	
Weak	4	1	1	0.028* 0.408
Strong	3	2	0	
Very strong	5	4	9	

a As long as the sample contained cells with the dishevelled binding antagonist of beta catenin 1 (DACT1) cytoplasmic staining, the staining was regarded as weak if mean density was <0.025, strong if it was between ≥0.025 and <0.04, and very strong if it was ≥0.04.

b As long as the sample contained cells with β-catenin cytoplasmic and/or membrane staining, the total expression level was regarded as weak if mean density was <0.01, strong if it was between ≥0.01 and <0.03, and very strong if it was ≥0.03. The nonparametric Spearman's rank correlation test was used for correlation analysis.

* *P*<0.05.

**Fig. 3:**
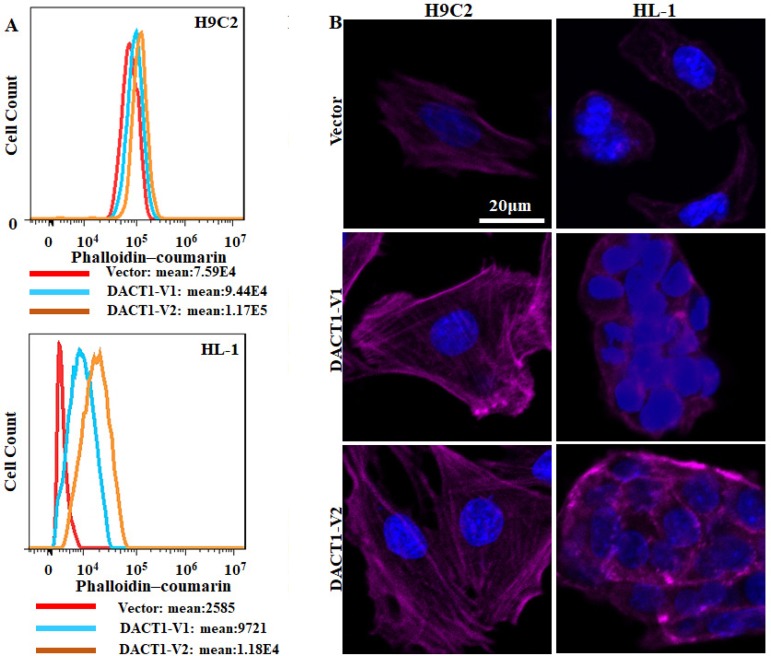
Effect of DACT1 on F-actin rearrangement. (A) Transduced cells were stained with phalloidin-coumarin, and the F-actin content was analyzed using flow cytometry (count number for cells ≥ 3000; the experiment was independently repeated at least twice). (B) Transduced cells were fixed, and the F-actin organization was analyzed by phalloidin staining and immunofluorescence. DACT1=dishevelled binding antagonist of beta catenin 1; F-actin=fibrous actin

The concentration and distribution of F-actin, an essential element of the cytoskeleton, were then detected. Phalloidin-coumarin staining indicated that DACT1 overexpression increased the F-actin concentration (Figure 3A). Furthermore, fluorescent images of vector groups showed less bundled actin in the vector groups than in the DACT1-V1 and DACT1-V2 groups in both H9C2 and HL-1 cells (Figure 3B). Taken together, these findings indicate that DACT1 might induce cytoskeletal rearrangement by altering the cytoskeletal proteins such as β-catenin in myocardial cells.

### The Regulation of the Cardiac Gap Junction Cx43 by DACT1

**Fig. 4:**
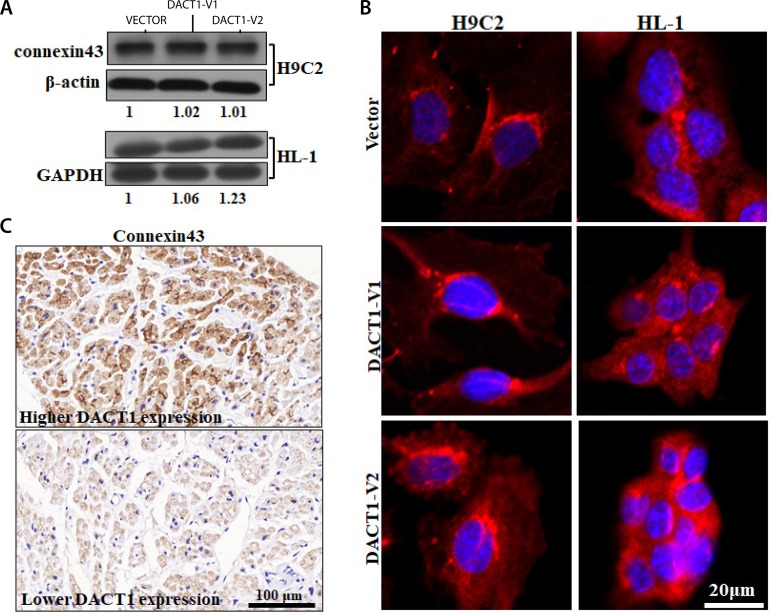
The effect of DACT1 on Cx43 in myocardial cells. The effect of DACT1 overexpression on Cx43 concentration A) and distribution B) in H9C2 and HL-1 cells. C) Cx43 expression in the myocardial tissues of patients with valvular heart disease. DACT1=dishevelled binding antagonist of beta catenin 1; GAPDH=glyceraldehyde-3-phosphate dehydrogenase

Given that cytoskeletal reorganization could lead to an alteration of expression and cellular distribution of Cx43 and gap junction remodeling^[20]^, we hypothesized that the Cx43 might be involved in the regulation of the cytoskeleton by DACT1 in myocardial cells. High Cx43 expression was associated with low fibrosis degree, right ventricular (RV), and RA values (Supplement Table 5), suggesting the structural and fibrotic relevance of Cx43. In the present study, overexpression of neither DACT1 isoform could alter Cx43 concentration in H9C2 and HL-1 cells (Figure 4A); however, an increase in the distributed heterogeneity was observed (Figure 4B), suggesting that a sole DACT1 isoform could affect only the Cx43 location in the myocardial cell. Interestingly, we found a significant association between DACT1 expression and Cx43 in the patients with valvular heart disease (P=0.048, Spearman’s rho=0.370) (Figure 4C, Supplement Table 6). We speculated that both the DACT1 isoforms existed in the clinical samples and that their synergistic effect might exist to regulate Cx43. Thus, their association could only be observed in the clinical samples.

**Supplement Table 5:** The correlation between the patients' clinical parameters and connexin 43 (Cx43) expression level in the myocardium of patients with valvular heart disease.

Indices	Negative	Very weak	Weak	Strong	Very strong	Spearman's rho *P*
EF (%)	62.000±8.803	63.928±7.721	58.000±0.000	60.833±6.014	64.000±7.810	0.036 0.855
LA (mm)	58.800±15.643	61.357±24.566	85.000±0.000	43.333±9.004	44.333±6.028	-0.323 0.087
LVD (mm)	50.200±9.757	53.143±10.347	57.000±0.000	55.000±9.166	44.000±9.000	0.015 0.939
LVS (mm)	32.400±3.912	33.928±5.916	39.000±0.000	36.833±6.969	28.667±7.637	0.068 0.724
IVS (mm)	9.400±1.817	10.036±1.623	7.000±0.000	9.667±2.251	11.000±3.000	-0.133 0.491
LVPW (mm)	8.800±0.837	9.857±1.406	9.000±0.000	9.333±2.066	11.000±1.000	0.128 0.508
RA (mm)	70.600±8.849	56.714±14.242	72.000±0.000	51.667±10.367	49.000±9.849	-0.429 0.020*
RV (mm)	24.600±2.074	28.714±13.070	20.000±0.000	22.000±3.033	20.000±3.606	-0.378 0.043*
Fibrosis degree	0.203±0.051	0.163±0.010	0.208±0.000	0.126±0.054	0.096±0.0149	-0.407 0.028*

As long as the sample contained cells with Cx43 cytoplasmic and/or membrane staining, the total expression level was regarded as negative if mean density was <0.01, very weak if it was between ≥0.01 and <0.03, weak if it was between ≥0.03 and <0.04, strong if it was between ≥0.04 and <0.05, and very strong if it was ≥0.05. Fibrosis degree was detected by Masson's staining. Fibrosis degree=the area of fibrosis/total area. The nonparametric Spearman's rank correlation test was used for correlation analysis.

* *P*<0.05.

EF=ejection fraction; IVS=interventricular septum; LA=left atrial; LVD=left ventricular diastolic; LVPW=left ventricular posterior wall; LVS=left ventricular systolic; RA=right atrial; RV=right ventricular

**Supplement Table 6:** The relationship between DACT1 expression and connexin 43 (Cx43) expression in the myocardium of patients with valvular heart disease.

Cx43 expression^b^	DACT1 expression^a^		*P* Spearman's rho
	Weak	Strong/Very strong	
Negative	2	3	0.048*
Very weak	4	10	0.370
Weak	0	1	
Strong	0	6	
Very strong	0	3	

a As long as the sample contained cells with the dishevelled binding antagonist of beta catenin 1 (DACT1) cytoplasmic staining, the staining was regarded as weak if mean density was <0.025, strong if it was between ≥0.025 and <0.04, and very strong if it was ≥0.04;

b As long as the sample contained cells with connexin 43 cytoplasmic and/or membrane staining, the total expression level was regarded as negative if mean density was <0.01, very weak if it was between ≥0.01 and <0.03, weak if it was between ≥0.03 and <0.04, strong if it was between ≥0.04 and <0.05, and very strong if it was between ≥0.05. The nonparametric Spearman's rank correlation test was used for correlation analysis.

* *P*<0.05.

## DISCUSSION

AF, the most common cardiac arrhythmia in clinical practice, is a major cause of embolic stroke^[21]^. Despite the recent significant advances in the understanding of the mechanisms associated with AF, complexities in the etiology of atrial electrical dysfunction have prevented definitive elucidation^[22]^.

DACT1, which was first found as a Dvl-interacting protein, plays a crucial role in the normal vertebrate development^[23]^ and human diseases^[14]^ by affecting β-catenin. It was proposed that DACT1 was very important in human diseases at least because of the contribution for determining the role of β-catenin, a structural protein and/or a signal transduction protein. Our investigation in the present study confirmed this hypothesis. In the myocardium of the patients, β-catenin was not expressed in the nucleus, but was present in the cytoplasm and/or membrane. Furthermore, its expression was positively correlated with DACT1 cytoplasmic expression, suggesting that DACT1 may facilitate the extranuclear accumulation of β-catenin, which might serve as a structural protein in the myocardial cells of the valvular heart disease patients. Further analysis of the myocardial structure confirmed this speculation. DACT1 induced F-actin filament accumulation in the myocardial cells. Actin filaments reorganization can lead to alteration of the gap junction protein Cx43^[24]^. Thus, unsurprisingly, we found a correlation between the expression of DACT1 and Cx43 in clinical samples and found that DACT1 altered the cellular distribution of Cx43 in the cardiac cell line. Cx43 is a very important susceptibility gene in AF, and the reduced number and organization of Cx43-containing gap junction plaques likely play a fundamental role in the increased incidence of arrhythmias and degree of fibrosis^[25,26]^. We also found the structural and fibrotic relevance of Cx43 (Supplement Table 5), and therefore, Cx43 remodeling may be the reason for the association of the weak DACT1 expression with AF and the degree of fibrosis found in our study. Thus, it is proposed that DACT1 is a novel AF-related gene by regulating Cx43 via β-catenin in the myocardial cells. To our knowledge, it was the investigation of the involvement of DACT1 with human AF.

## CONCLUSION

In summary, we demonstrated for the first time that a lack of DACT1 was associated with AF and the degree of fibrosis in valvular heart disease. Specifically, DACT1 regulated the gap junction protein Cx43 by accelerating the cytoskeleton rearrangement via accumulation of β-catenin in the myocardial cells, indicating that new mechanisms mediated by DACT1 exist in AF. DACT1 is a novel potential therapeutic target for AF.

**Table:** 

Authors' roles & responsibilities	
JH	The acquisition, analysis, or interpretation of data for the work; final approval of the version to be published
YY	The acquisition, analysis, or interpretation of data for the work; final approval of the version to be published
BH	The acquisition, analysis, or interpretation of data for the work; final approval of the version to be published
GX	Drafting the work or revising it critically for important intellectual content; final approval of the version to be published
RS	Drafting the work or revising it critically for important intellectual content; final approval of the version to be published
LL	Drafting the work or revising it critically for important intellectual content; final approval of the version to be published
JH	The acquisition, analysis, or interpretation of data for the work; final approval of the version to be published
JY	Drafting the work or revising it critically for important intellectual content; final approval of the version to be published
YG	The acquisition, analysis, or interpretation of data for the work; final approval of the version to be published
KW	The acquisition, analysis, or interpretation of data for the work; final approval of the version to be published
ZW	Agreement to be accountable for all aspects of the work in ensuring that questions related to the accuracy or integrity of any part of the work are appropriately investigated and resolved; final approval of the version to be published
